# Koebner phenomenon induced by the use of a computer mouse in an occupational setting: case report

**DOI:** 10.47626/1679-4435-2020-599

**Published:** 2020-12-11

**Authors:** Miguel Alpalhão, Joana Antunes, Luís Soares-Almeida, Paulo Filipe

**Affiliations:** 1 Departamento de Dermatologia, Centro Hospitalar Universitário Lisboa Norte - Lisboa (LIS), Portugal; 2 Clínica Universitária de Dermatologia, Faculdade de medicina, Universidade de Lisboa - Lisboa (LIS), Portugal; 3 Unidade de Investigação em Dermatologia, Instituto de Medicina Molecular João Lobo Antunes - Lisboa (LIS), Portugal

**Keywords:** case report, occupational dermatosis, psoriasis

## Abstract

Occupational activities are well-known triggers for the onset or aggravation of several dermatoses. The Koebner phenomenon is characterized by the appearance of cutaneous lesions typical of a given inflammatory dermatosis in an area where the skin was injured by mechanical, chemical, or biological agents. Although it is usually easily identified when associated to significant trauma, the Koebner phenomenon may go unnoticed when a small-scale injury underlies its pathogenesis. Herein, we report a case of Koebner phenomenon induced by the repetitive use of a computer mouse in an occupational setting, leading to recalcitrant psoriatic lesions on the palm of the right hand. When atypical features or unexpected poor responses to treatment are observed in skin conditions, a complete social and occupational anamnesis is paramount to identify aggravating factors and allow successful patient management.

## INTRODUCTION

The Koebner phenomenon is characterized by the appearance of cutaneous lesions typical of a given inflammatory dermatosis in an area where the skin was injured by mechanical, chemical, or biological agents. The triggering trauma is usually identified as an acute and time-limited event, but chronic trauma due to occupational activities may also elicit this phenomenon.

## CASE REPORT

We present the case of a 35-year-old White male patient, with a Fitzpatrick III skin phototype, who works as a software developer. He was diagnosed with plaque-type psoriasis 18 years ago and with psoriatic arthritis 10 years ago; treatment was performed with topical agents, as well as oral methotrexate, with good disease control (Psoriasis Area Severity Index score below 5). However, the patient has experienced progressive thickening and scaling of the palm of his right hand for the last 6 years, with no lesions on the left hand. The patient did not have any complaints of pain, itching, or pustules, and reported that these lesions persisted even when no other psoriatic lesions were found on his body; the lesions were recalcitrant to all treatment attempts.

**Figure 1:**
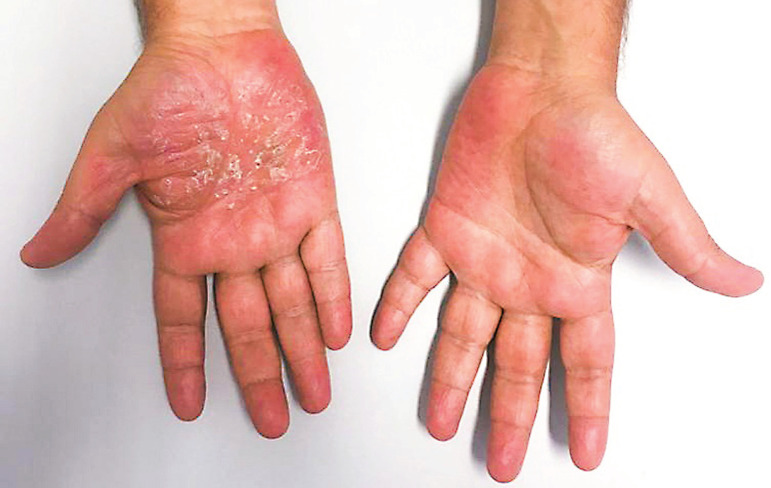
Clinical picture of both hands. Erythematous hyperkeratotic patch with micaceous scaling is seen on the palm of the right hand. No alterations are seen on the left hand.

Upon clinical observation, a plaque of epidermal thickening was seen on the palmar region of the right hand with well-defined borders, but no finger involvement. This plaque featured a reddish-violaceous coloration and micaceous scaling. The observation of the left hand was unremarkable (Figure 1).

A skin biopsy was performed and showed hyperkeratosis with parakeratosis, hypogranulosis, and intracorneal pustules; these aspects were suggestive of psoriasis.

## DISCUSSION

**Figure 2:**
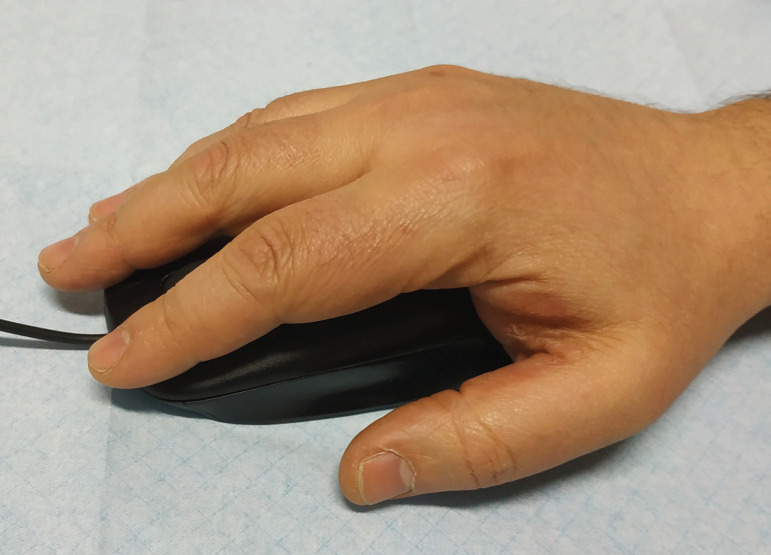
Patient holding the computer mouse. The contact area fully matches the lesion in the right palm.

These aspects were interpreted as the Koebner phenomenon induced by occupational activity. During his professional duties, the patient spends more than 10 hours daily using a computer and moving the computer mouse with his right hand. Indeed, the area of palmar thickening and scaling fully matched the surface that rested on the computer mouse (Figure 2).

This case illustrates a well-known phenomenon in psoriasis that is not usually seen in the context of occupational activities involving desk work. Nevertheless, a chronic and repetitive small-scale trauma such as the one induced by regular mouse use over long periods of time can elicit the Koebner effect in predisposed individuals.^1^

The unusual presentation was itself the clue that led to diagnosis: The differential signs of the disease between two symmetrical anatomical areas and the progressive worsening of the palmar complaints despite optimal control of psoriasis activity in the remaining tegument suggested local continuous exposure to an aggravating factor.^2^

In conclusion, in the presence of unusual clinical presentations, it is our duty to collect complete medical and social histories in order to identify potential aggravators of a given dermatosis. Occupational activities should be explored in detail, as repetitive or prolonged exposures to environmental aggressors in this setting may be decisive in an unsatisfactory response to treatment.

## References

[1] Samitz MH (1985). Repeated mechanical trauma to the skin: occupational aspects. Am J Ind Med.

[2] Ji YZ, Liu SR (2019). Koebner phenomenon leading to the formation of new psoriatic lesions: evidences and mechanisms. Biosci Rep.

